# The influence of kinesiology tape colour on performance and corticomotor activity in healthy adults: a randomised crossover controlled trial

**DOI:** 10.1186/s13102-018-0106-4

**Published:** 2018-11-01

**Authors:** Rocco Cavaleri, Tribikram Thapa, Paula R. Beckenkamp, Lucy S. Chipchase

**Affiliations:** 1 Brain Rehabilitation and Neuroplasticity Unit, School of Science and Health, Western Sydney University, Sydney, NSW 2560 Australia; 2 Musculoskeletal Health, Faculty of Health Sciences, Discipline of Physiotherapy, The University of Sydney, Sydney, NSW Australia; 3 Faculty of Health, University of Canberra, Canberra, ACT Australia

**Keywords:** Kinesiology tape, Colour, Performance, Corticomotor activity, Transcranial magnetic stimulation

## Abstract

**Background:**

There exists conflicting evidence regarding the impact of kinesiology tape on performance and muscle function. One variable that may account for disparities in the findings of previous studies is the colour of the tape applied. Colour is hypothesised to influence sporting performance through modulation of arousal and aggression. However, few studies have investigated the influence of colour on products designed specifically to enhance athletic performance. Further, no studies have investigated the potential influence of colour on other drivers of performance, such as corticomotor activity and neuromuscular function. Thus, the aim of this study was to investigate the influence of kinesiology tape colour on athletic performance, knee extensor torque, and quadriceps neuromuscular function.

**Methods:**

Thirty two healthy participants were assessed under five conditions, applied in random order: (1) no kinesiology tape (control), (2) beige-coloured kinesiology tape applied with tension (sham A), (3) beige-coloured kinesiology tape applied with no tension (sham B), (4) red-coloured kinesiology tape applied with tension, and (5) blue-coloured kinesiology tape applied with tension. Athletic performance was assessed using a previously validated hop test, knee extensor torque was measured using an isokinetic dynamometer, and transcranial magnetic stimulation was utilised to provide insight into the neuromuscular functioning of the quadriceps musculature.

**Results:**

Kinesiology tape had no beneficial impact on lower limb performance or muscle strength in healthy adults. The colour of the tape did not influence athletic performance (F (4, 120) = 0.593, *p* = 0.669), quadriceps strength (F (4, 120) = 0.787, *p* = 0.536), or neuromuscular function (rectus femoris: F (2.661, 79.827) = 1.237, *p* = 0.301).

**Conclusion:**

This study found that kinesiology tape does not alter lower limb performance or muscle function in healthy adults, irrespective of the colour of the tape applied. Future research should seek to confirm these findings beyond the research setting, across a range of sports, and at a range of skill levels.

**Trial registration:**

Australian New Zealand Clinical Trials Registry. ACTRN12616001506482. Prospectively registered on 01/11/2016.

## Background

Maximising performance and facilitating recovery following injury are important goals for athletes and their rehabilitation providers [1]. There is an ever-expanding range of products designed to assist with achieving these goals [1, 2]. One such product, kinesiology tape, is being used increasingly by athletes seeking a physiological or psychological advantage [1, 2]. Kinesiology tape has been proposed to enhance performance by supporting musculature, joints, and fascia without limiting range of motion [2, 3]. Additionally, kinesiology tape is believed to promote healing by facilitating lymphatic drainage, blood circulation, and corticomotor activity [4–6].

Although the use of kinesiology tape has increased dramatically since it was first developed in the 1980s [5], there is uncertainty regarding its effectiveness [7, 8]. Recent systematic reviews have found moderate evidence that kinesiology tape does not improve recovery or pain following musculoskeletal injury [7, 8]. These findings suggests that the near-ubiquitous use of kinesiology tape in professional sport may be due to a perceived advantage in terms of performance, rather than recovery [7, 9]. However, research into the effect of kinesiology tape on performance is conflicting [9]. While a number of studies report that kinesiology tape positively influences athletic performance, lower limb strength, and neuromuscular function [10–14], others report that kinesiology tape has no effect on these outcomes [15–19]. Despite this uncertainty, user satisfaction with kinesiology tape remains high, suggesting that there may be a psychological component associated with its use [7].

A psychological factor that may account for disparities in the findings of previous studies is the effect of the colour of the tape applied. Several studies provide evidence of a relationship between colour and sporting success. For example, when individual skill levels were taken into account, Hill and Barton [20] demonstrated that contestants in red uniforms had a significantly greater chance of victory in bouts of Olympic boxing, taekwondo, freestyle wrestling, and Greco-Roman wrestling compared to those in blue uniforms. This ‘red advantage’ has also been documented in team sports including rugby league [21], and has been linked to long-term success in the English Premier League [22]. Indeed, the colour red has been proposed to enhance strength output and neuromuscular function in simple motor tasks via a threat-based response, thought beneficial during short bursts of activity [20]. Conversely, exposure to the colour blue has been linked with enhanced performance during creative tasks, and is thought to promote relaxation [23]. Such findings hold particular implications for kinesiology tape, which is often worn to enhance explosive performance [7].

While the colour red has been linked with sporting success, there are conditions in which wearing or viewing red may be counterproductive. Elliot & Aarts [24] proposed that the threat-based response associated with the colour red may be a ‘double-edged sword’, enhancing performance in some contexts but leading to distraction, fatigue, and worry in others. This hypothesis is supported by evidence indicating that students perform worse when exposed to the colour red before attempting an exam [25]. There is also evidence to suggest that the ‘red advantage’ may actually be due to a decrease in performance among the opposition, rather than an increased performance among the athletes wearing red themselves [20]. The influence of colour may therefore be context-specific, affected by the purpose and duration of exposure [20].

Multiple factors, ranging from an athlete’s choice of footwear [26] to the colour of their jersey [20–22], have the potential to tip the balance in a close sporting contest. However, few studies have investigated the influence of colour on products designed to enhance athletic performance. Further, no studies have investigated the potential influence of colour on other drivers of performance, such as corticomotor activity and neuromuscular function. Kinesiology tape represents a valuable means by which to explore the role of colour in these contexts. Therefore, the aim of this study was to investigate the influence of kinesiology tape colour on athletic performance, strength, and neuromuscular function among healthy adults.

## Methods

### Study design

A randomised crossover controlled trial was conducted in the School of Science and Health at Western Sydney University in New South Wales, Australia. Reporting of the study followed the Consolidated Standards of Reporting Trials (CONSORT) guideline extension for crossover trials [27], as well as the recommendations provided by Li, Yu, Hawkins and Dickersin [28]. The study was funded by the School of Science and Health at Western Sydney University.

### Ethics and registration

All participants received written and verbal descriptions of the experiment and provided written informed consent prior to testing. The study was conducted in accordance with the Declaration of Helsinki [29] and Australia’s National Statement on Ethical Conduct in Human Research [30]. This study was approved by the Human Research Ethics Committee at Western Sydney University (H10184), and was registered with the Australian New Zealand Clinical Trials Registry (ACTRN12616001506482).

### Recruitment and eligibility

Healthy participants aged between 18 and 40 years were eligible for the study. Healthy individuals were recruited to ensure that any changes in athletic performance, strength, or neuromuscular function could be attributed to the colour of the tape applied rather than the provocation of pain or other symptoms. Participants were recruited through flyers and advertisements on social media networks. People were excluded if they experienced pain during data collection or presented with lower limb osteoarthritis or rheumatoid arthritis; low back pain; referred or radicular pain to the lower limb in the previous 12 months; sensory disturbances; a history of ankle or knee instability; a history of osteomyoarticular lesion or surgery in the lower limb over the last 12 months; non-corrected neurological, vestibular, visual or hearing impairments; or allergy to adhesive material. All participants were also safety screened for transcranial magnetic stimulation (TMS) to exclude individuals with contraindications to TMS or a history of stroke, seizure, epilepsy, brain injury, metallic implants, and use of implanted devices [31].

### Intervention

**Fig. 1:**
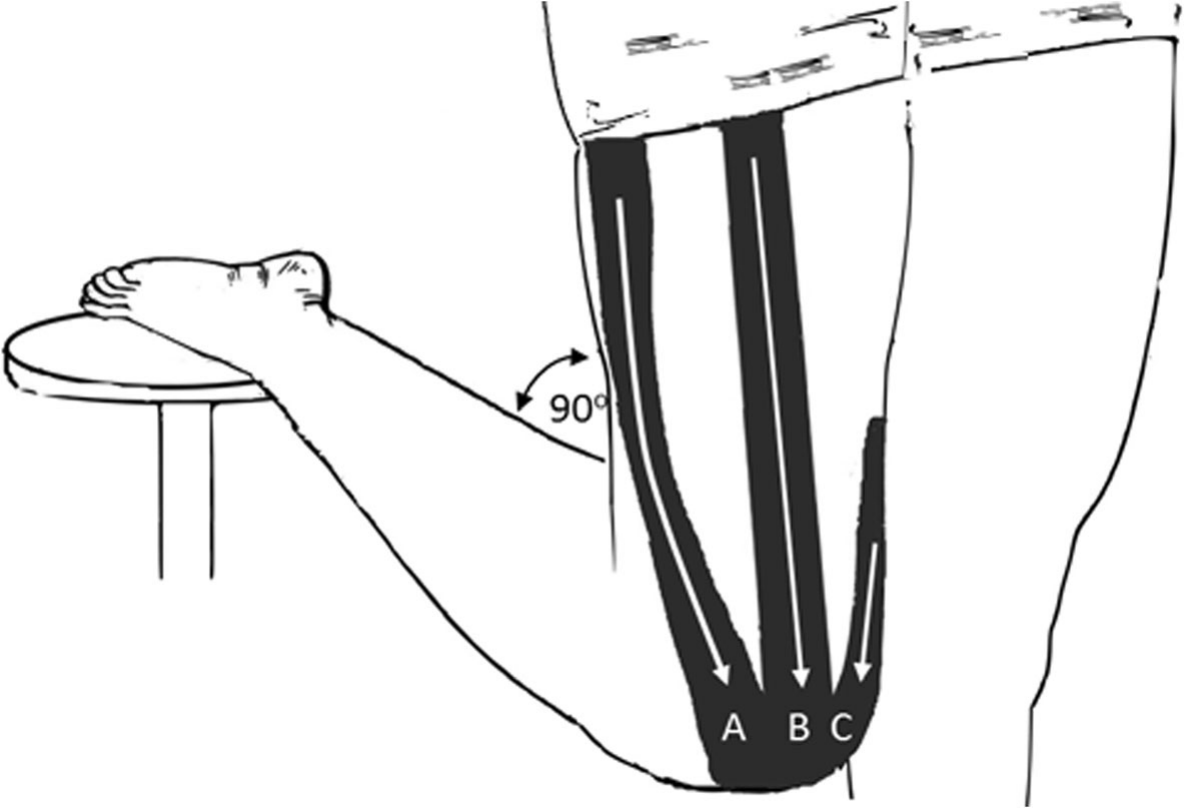
Application of kinesiology tape. **a**: Tape applied over vastus lateralis, from the greater trochanter to the lateral boarder of the patella. **b**: Tape applied over rectus femoris, from 10 cm below the anterior superior iliac spine to the superior boarder of the patella. **c**: Tape applied over vastus medialis, from the middle third of vastus medialis to the medial boarder of the patella. Tape was applied with 50% tension. Figure created by the author

**Fig. 2:**
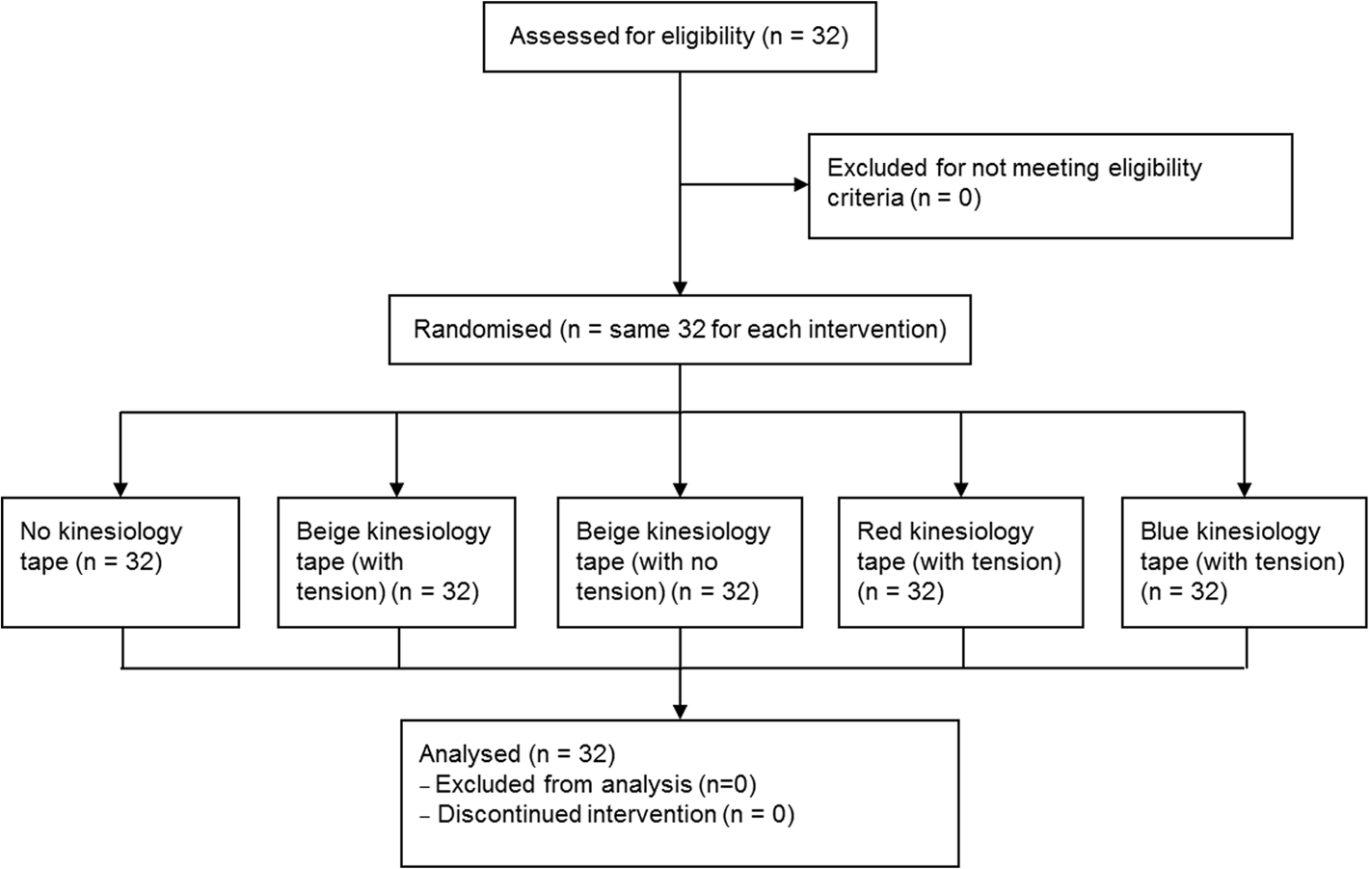
Flow of participants through study

All participants were assessed under five experimental conditions, allocated in random order: (1) no kinesiology tape (control), (2) beige-coloured kinesiology tape applied with tension (sham A), (3) beige-coloured kinesiology tape applied with no tension (sham B), (4) red-coloured kinesiology tape applied with tension, and (5) blue-coloured kinesiology tape applied with tension (Team Tape®) (see Fig. 1 and Fig. 2).

Randomisation was concealed using opaque envelopes and conducted by an independent person not involved in data collection. For conditions involving kinesiology tape application (red, blue, or beige), the tape was applied to the rectus femoris, vastus lateralis, and vastus medialis muscles of the dominant lower limb. Longitudinal strips of the tape were applied from proximal to distal with 50% tension in the middle (apart from the no tension condition), and no tension at the end of the strip [17]. This approach has been recommended previously [5, 17]. Kinesiology tape on the rectus femoris muscle was applied from 10 cm below the anterior superior iliac spine to the superior boarder of the patella [17]. Tape applied to the vastus lateralis muscle extended from the greater trochanter to the lateral boarder of the patella, while tape on the vastus medialis muscle extended from the middle third of the vastus medialis muscle to the medial boarder of the patella [17]. When applying the tape, all participants were asked to stand on the non-dominant leg, while flexing the dominant knee at 90 ^o^ with support (see Fig. 1). Positioning the knee in flexion and applying the tape from proximal to distal is consistent with previous investigations and is thought to facilitate quadriceps activation [7, 8, 32, 33].

### Outcomes

The primary outcome was lower limb functional performance (distance on the single leg hop test) and secondary outcomes included: (1) knee extensor torque (maximal torque produced using an isokinetic dynamometer); and (2) neuromuscular function of the quadriceps muscles (assessed using transcranial magnetic stimulation). Participant characteristics such as age, gender, leg dominance (right or left), leg length (distance from the anterior superior iliac crest to medial malleolus), body mass index (BMI, weight/height^2^), and participant colour preference were also collected.

#### Lower limb functional performance

Lower limb functional performance was assessed with the single leg hop test, a reliable measure of the strength and functional stability of the lower limb [17, 34]. After a verbal explanation of the test, participants were asked to hop horizontally as far as possible using only the dominant limb. All participants were asked to perform the test barefoot. Hallux-hallux distance (cm) was recorded using a metric tape, and the greatest distance of three attempts was recorded [17, 34].

#### Knee extensor torque

Knee extensor torque was obtained using an isokinetic dynamometer. When recording knee extensor torque, subjects were seated with their hips and dominant thigh supported, and the pelvic and thoracic regions secured by a belt. The force pad of the dynamometer was positioned 5 cm above the centre of a line drawn between the distal ends of the medial and lateral malleoli [17]. Isometric knee extensor activity was assessed with the knee flexed at 90^o^. Standardised verbal encouragement was provided throughout the assessment to all participants. The best of three performances was recorded for each experimental condition.

#### Neuromuscular function

Following assessment of knee extensor torque, transcranial magnetic stimulation (TMS) was used to explore the corticomotor activity of the quadriceps muscle. Corticomotor activity was used as an index of neuromuscular function [35]. Assessments were performed during active voluntary contraction of the quadriceps. Participants were seated in a chair with their head and arms supported. Resisted knee extension at 10% of the participant’s maximal voluntary contraction (MVC) was performed when recording corticomotor activity [36]. Real-time visual feedback was provided on a computer screen in the form of electromyography recordings. All data was obtained from single-pulse, monophasic stimuli delivered using a figure-of-eight coil connected to a Magstim 200 stimulator (Magstim Co. Ltd., Dyfed, UK). The optimal cortical site for stimulation (‘hotspot’) was determined by identifying the coil position that evoked a maximal peak-to-peak motor evoked potential (MEP) in the rectus femoris muscle [36]. Active motor threshold (aMT) was then determined at the hotspot. The aMT was defined as the minimum TMS intensity required to elicit at least five discernible MEPs in a trail of ten stimuli at 10% MVC of the quadriceps muscle [35–39]. Stimulation intensity for the rest of the testing session was then set at 110% of aMT [37–39]. Electromyography recordings were obtained using Ag-AgCl electrodes (Noraxon dual electrodes, product #272S, inter-electrode distance 2.0 cm) positioned over the muscle belly of the rectus femoris, vastus lateralis, and vastus medialis muscles. Twenty peak-to-peak MEP amplitudes were recorded and stored on a computer for offline analysis using Signal software (version 5.08 × 86, Cambridge Electronic Design, Cambridge, UK).

#### Participant colour preference

After testing, participants were questioned regarding their colour preference when presented with the options of ‘red’, ‘blue’, and ‘no preference’. Colour preference was collected as it is hypothesised to be linked with arousal levels, personality traits, and athletic performance [40–43].

### Data processing and storage

Participants were de-identified using codes generated by the researchers. Assessments and kinesiology tape application were conducted by two registered physiotherapists. To minimise human error, collected data were entered into two separate spreadsheets by two of the researchers. These spreadsheets were then compared, with any discrepancies being investigated and corrected. All hard copies were stored in a locked filing cabinet and digital copies were stored in password-protected computers.

### Statistical analysis

Data analyses were performed using SPSS (version 23.0; IBM, New York). Two-way repeated measures analyses of variance (ANOVAs) were performed to analyse hop distance, knee extensor torque, and corticomotor activity with the factors “taping condition” and “colour preference”. Assumptions of normality and sphericity (equal variance) for parametric analyses were assessed using the Shapiro-Wilk test and Mauchly’s test of sphericity, respectively. The Greenhouse-Geisser correction was applied for data sets that violated the assumption of sphericity [44]. To detect a mean difference of 5 cm on the single leg hop test between the red and blue tape conditions (80% power), a total of 32 participants was required [34]. An intention-to-treat principle was used for data analyses, and those analysing the data were blinded to taping conditions and the nature of the study.

## Results

Thirty two healthy, right-leg-dominant individuals (16 females, mean ± standard deviation [SD] age of 24 ± 5 years) participated. The mean ± SD participant BMI and lower limb length were 23.3 ± 2.8 and 79.48 ± 5.82 cm, respectively. Overall, thirteen participants preferred the colour blue and nineteen participants preferred the colour red. Figure 2 shows the flow of participants through the study.

### Lower limb functional performance

There was no significant interaction between colour preference and taping condition in terms of lower limb functional performance (F (4, 120) = 1.485, *p* = 0.211). The main effect of taping condition was also not significant (F (4, 120) = 0.593, *p* = 0.669). There was no significant difference in performance between those who preferred red and those who preferred blue (F (1, 30) = 2.714, *p* = 0.110). These results indicate that lower limb functional performance was not influenced by tape colour, amount of tension on the tape, or personal colour preferences.

### Knee extensor torque

No significant interaction was identified between colour preference and taping condition for knee extensor torque (F (4, 120) = 1.379, *p* = 0.245). The main effect of taping condition was also not significant (F (4, 120) = 0.787, *p* = 0.536). There were no significant differences overall between those who preferred red and those who preferred blue in terms of knee extensor torque recordings (F (1, 30) = 0.469, *p* = 0.499). These results indicate that knee extensor torque was not influenced by tape colour, amount of tension on the tape, or personal colour preferences.

### Neuromuscular function

There were no significant interactions between colour preference and taping condition on motor evoked potential amplitude (reflecting corticomotor activity and neuromuscular function) for the rectus femoris (F (2.661, 79.827) = 0.598, *p* = 0.599), vastus medialis (F (2.336, 70.074) = 0.305, *p* = 0.771), or vastus lateralis (F (2.405, 72.140) = 1.311, *p* = 0.277). The main effect of taping condition was also not significant for any of these muscles (RF: F (2.661, 79.827) = 1.237, *p* = 0.301), VM: (F (2.336, 70.074) = 0.408, *p* = 0.698), VL: (F (2.405, 72.140) = 0.887, *p* = 0.433). Overall, colour preference did not influence neuromuscular function (RF: F(1, 30) = 2.752, *p* = 0.108), VM: F(1, 30) = 0.486, *p* = 0.491), VL: (F(1, 30) = 0.207, *p* = 0.652). These results indicate that neuromuscular function was also not influenced by tape colour or the amount of tension applied to the tape.

## Discussion

This study provides evidence that the colour of kinesiology tape has no effect on athletic performance, muscle strength, or neuromuscular function in healthy adults. In addition, our work supports previously published data in this population group indicating that kinesiology taping is not effective when compared to no tape or tape applied without tension. No psychological or neurophysiological mechanisms supporting the application of kinesiology tape to improve performance in healthy individuals were identified.

Different colours have been shown previously to have distinct impacts on emotion, performance, and aggression [20, 22, 25]. In fact, research in individual and team sports suggests that jersey colour can influence success rates in evenly matched contests, with red being more strongly associated with winning than other colours [20–22]. The colour red is also thought to increase the force of motor output and the speed with which force is generated [20].

Our findings that the colour of kinesiology tape had no effect on muscle strength conflict with previous research indicating that the colour red enhances force generation and motor output [20]. There are a number of plausible explanations for this discrepancy. For example, previous studies have presented the colour red to participants in a static manner and instructed them to view the colour directly [20, 24, 25]. In our study, participants were not instructed specifically to view their tape application, and therefore may have been less influenced by colour differences. Further, the amount of tape applied may not have been sufficient to produce a colour effect. Participants were also wearing shorts and t-shirts during testing and, as the colour of these garments was not controlled, this may have minimised the effect of tape colour on performance.

Conversely, the lack of a significant difference between colours may be due to the notion that viewing red results in a decrease in opposition performance, rather than an increased performance of the wearer themselves [20, 24]. For example, Recours & Briki [45] found that participants exposed to an opponent in a blue uniform reported high levels of self-confidence, while those exposed to an opponent in red reported higher levels of cognitive anxiety. There is also research indicating that viewing red stimuli is associated with impaired performance, and that this effect takes place outside of participants’ conscious awareness [46]. This is thought to be due to the colour red’s association with the danger of failure in achievement contexts and evocation of avoidance motivation [46]. The present study provides initial evidence that red-perceptions do not translate to functional differences without a face-to-face opponent. A useful follow-up study would be to examine the effect of the colour of kinesiology tape on perceptions and performance of an ‘opposition’ participant.

Our findings also suggest that kinesiology tape is not effective at improving performance in healthy individuals, irrespective of colour. This supports previous work that has demonstrated kinesiology tape application does not improve muscle strength or function in this population [15–17]. In addition, our work concurs with previous data demonstrating that kinesiology taping conditions have no effect on motor-evoked potential amplitudes in calf musculature at rest or during movement, highlighting that this is also the case in muscles of the thigh [6]. Taken together, these data suggest that kinesiology tape application is unlikely to modulate neuromuscular activity among healthy individuals in a functionally useful manner.

While systematic reviews report insufficient evidence to support the use of kinesiology tape following musculoskeletal injury, and there are conflicting reports on the effect of the tape on performance, the use of kinesiology tape remains commonplace [7, 9]. In our randomised crossover controlled trial, no effect of kinesiology tape was found under any condition, whether it be due to placebo (tension versus non-tension) or not. Participant colour preferences also did not influence any of the results. The use of kinesiology tape is therefore likely associated with perceived, rather than true, benefits on performance. The aesthetic appeal of kinesiology tape could also be a factor contributing to its widespread use in athletics and requires further investigation.

Despite a rigorous approach towards data collection and analysis, this study is not without limitations. The research was laboratory-based and may not reflect real-world contexts involving competitors or audiences. Further research is required in more functionally meaningful contexts across a range of sports and at a range of skill levels. Additionally, the study was conducted in healthy individuals. As such, there may have been a ceiling effect in terms of performance improvement. Investigations involving athletes with pain or injuries are warranted to determine if the influence of kinesiology tape colour is modulated by these factors. As the primary variable of interest in this study was the colour of the tape applied, the brand of kinesiology tape is unlikely to have influenced the results. However, as subtle differences in mechanical properties have been observed between brands of kinesiology tape [47], additional research involving a broader variety of tape manufacturers, patterns, tensions, and colours is also warranted.

## Conclusions

This study found that kinesiology tape had no beneficial effects on athletic performance, muscle strength, or neuromuscular function, irrespective of the colour of the tape applied. The findings of this study add to the body of knowledge on the effectiveness of kinesiology tape and the impact of colour on performance. The study was a robust crossover controlled trial with random condition allocation to mitigate potential learning or fatigue effects on performance. However, further research is required to determine if the findings of this study are applicable beyond the laboratory setting and across a range of sporting contexts.

## Abbreviations

aMT: Active motor threshold
ANOVA: Analysis of variance
BMI: Body mass index
MEP: Motor evoked potential
MVC: Maximal voluntary contraction
TMS: Transcranial magnetic stimulation
